# Supplementary material for: Heteroatom-Enhanced Porous Carbon Materials Based on Polybenzoxazine for Supercapacitor Electrodes and CO2 Capture
Source: Polymers (Basel). 2023 Mar 21;15(6):1564. doi: 10.3390/polym15061564 (PMC10051936; doi:10.3390/polym15061564)
Supplement: Supplementary file 1 [file polymers-15-01564-s001.zip › polymers-2248793-supplementary.pdf]

Heteroatom-Enhanced Porous Carbon Materials Based on Polybenzoxazine for Supercapacitor Electrodes and CO_2 Capture

S1. Materials

Eugenol and paraformaldehyde were purchased from Sigma-Aldrich (USA). Ethanol, melamine, potassium hydroxide (KOH), N-methyl-2-pyrrolidone (NMP), sulphuric acid (H2SO4), polyvinylidene fluoride (PVDF), and sodium hydroxide (NaOH) were purchased from Duksan Chemicals Co., Ltd. Republic of Korea. Plain carbon cloth (bare CC) was purchased from FuelCellsEtc (USA). All chemicals were used without further purification.

S2. Instrumentation methods

The synthesized benzoxazine monomer were thoroughly characterized by various physicochemical techniques. Fourier transform infrared (FT-IR) spectra were obtained with a Perkin Elmer MB3000 FTIR spectrometer. The spectra were obtained at a resolution of 4 cm−1 in the IR range of 400–4000 cm−1. Samples were prepared by grinding with KBr and compressed to form discs. Nuclear magnetic resonance (NMR) spectra were recorded by using an Agilent NMR, VNS600 at a proton frequency of 600 MHz for 1H-NMR. Solutions were prepared by dissolving the samples in DMSO-d6. Prepared HCPCs were thoroughly characterized by various physicochemical techniques. X-ray diffraction (XRD) measurements were done with a PANalytical X'Pert3 MRD diffractometer using monochromatized Cu Kα radiation (λ = 1.54 Å) at 40 kV and 30 mA and were noted in the range from 10 to 90° (2θ). The dry powder was spread on top of a glass substrate and was then noted in reflection geometry. Raman spectrum was recorded on a Thermo Scientific DXR Smart Raman spectrometer with the range from 50 to 3000 cm−1. X-ray photoelectron spectroscopy (XPS) was performed using a K-Alpha (Thermo Scientific). The non-monochromatic Al Kα line at 180–200 W was used as the primary excitation. Casa XPS instrument software was used for the deconvolution of the high-resolution XPS spectrum. Field emission scanning electron microscopy (FESEM) images were observed on a Hitachi S-4800 equipped with energy-dispersive X-ray (EDX) spectrometer at an accelerating voltage of 10 kV. A small amount of sample solution was first drop cast on an ultrasonically cleaned Si-wafer substrate letting the solvent to evaporate. TEM/HRTEM images were performed with an FEI-Tecnai TF-20 transmission electron microscope with an operating accelerating voltage of 120 kV.

S3. Fabrication of working electrode and electrochemical measurements

The prepared HCPCs materials were used for the fabrication of the working electrode. To fabricate the working electrode, NRPCs materials, and polyvinylidene fluoride (PVDF) with the wt. % of 95:5 was ground well in an appropriate amount N-Methyl-2-Pyrrolidone (NMP) to make a homogeneous paste, respectively. The resulting homogeneous paste was coated on the conducting substrate (carbon cloth-CC) with an area of 1 cm^2 by the drop-casting method and sequentially the electrode was kept at 100 °C in a hot air oven for 48 h to dry the electrodes. After the fabrication, the obtained modified working electrodes were examined for supercapacitor activity. All electrochemical measurements including cyclic voltammetry (CV), galvanostatic charge-discharge (GCD), and electrochemical impedance spectroscopy (EIS) were conducted with a typical three-electrode system and were performed on the CorrTest-CS350 electrochemical workstation in 1 M H_2SO_4 aqueous solution. A commercial Hg/$HgSO_4$ (Sat. K_2SO_4) electrode was employed as a reference electrode, a platinum plate (1 cm^2) was used as a counter electrode and

HCPCs material loaded CC (HCPCs/CC) was used as working electrodes. The CV measurements were carried out at a potential window from 0.0 and 0.6 V (*vs*. $Hg/HgSO_4$). The GCD measurements were performed with a potential window of 0.0–0.6 V (*vs*. $Hg/HgSO_4$) at the current densities varied from 0.5 to 5 A g^{-1}. EIS measurements were performed in the frequency range of 0.01 kHz–100 kHz with an alternating current amplitude of 5 mV. All the electrochemical tests were conducted at room temperature. The capacitances of the electroactive materials were obtained from their GCD curves according to the following equation (S1).

$$C_s = \frac{I\Delta t}{m\Delta V} \quad \text{(S1)}$$

whereas, C_s is the specific capacitances (F g^{-1}), I is the current in the charge-discharge process (A), Δt represents to the discharge time (s), ΔV stands for the potential window during the charge-discharge measurement (V) and m donates the mass of the electroactive materials (g).

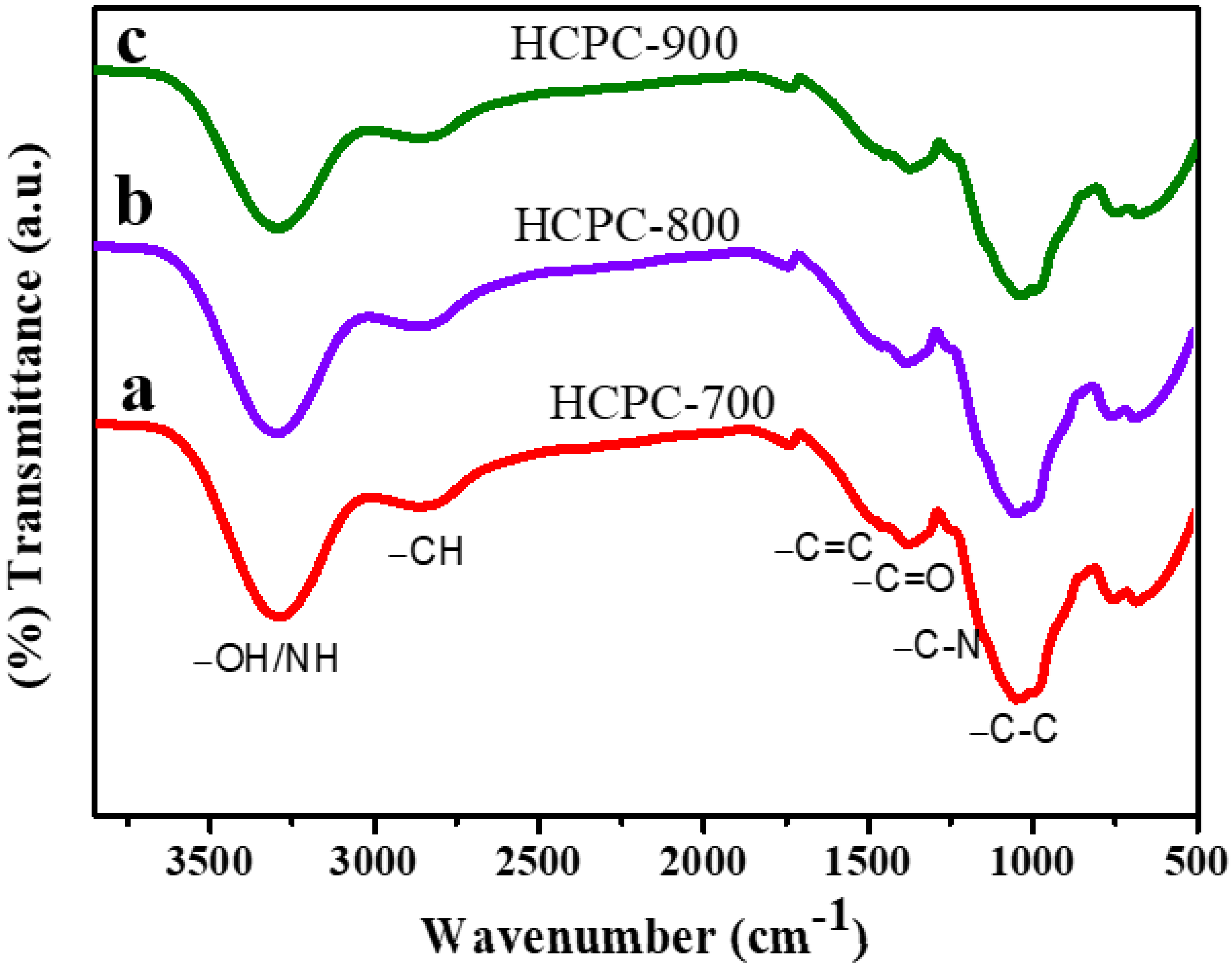

Figure S1. FT-IR spectra of (a) HCPC-700 (b) HCPC-800, and (c) HCPC-900.

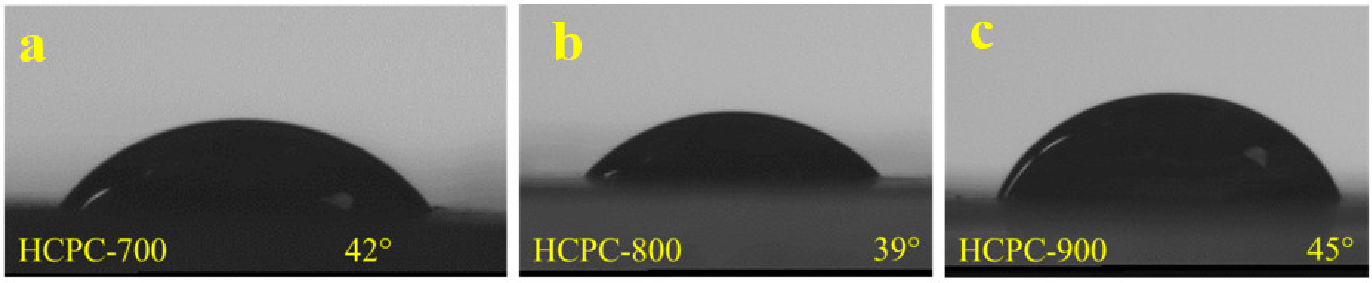

Figure S2. Contact angle images of (a) HCPC-700 (b) HCPC-800, and (c) HCPC-900.

Table S1. Comparison of CO_2 uptake at 0°C.

Adsorbents	CO2 uptake (mmol g^{-1})	Reference
Nitrogen enriched porous carbon	2.25	1
Chitosan derived nitrogen-doped microporous carbons	3.9	2
N-doped carbon from polypyrrole	3.9	3
NPC-2 derived from polybenzoxazine	4.02	4

References

1. Pevida, C.; Drage, T.C.; Snape, C.E. Silica-templated melamine–formaldehyde resin derived adsorbents for CO_2 capture. *Carbon* **2008**, 46, 1464–1474.
2. Fan, X.Q.; Zhang, L.X.; Zhang, G.B.; Shu, Z.; Shi, J.L. Chitosan derived nitrogen-doped microporous carbons for high performance CO_2 capture. *Carbon* **2013**, 61, 423–430.
3. Sevilla, M.; Vigon, P.V.; Fuertes, A.B. N-doped polypyrrole-based porous carbons for co_2 capture. *Adv. Funct. Mater.* **2011**, 21, 2781–2787.
4. Wan, L.; Wang, J.; Feng, C.; Suna, Y.; Li, K. Synthesis of polybenzoxazine based nitrogen-rich porous carbons for carbon dioxide capture. *Nanoscale* **2015**, 7, 6534–6544.
